# Lateral Dislocation of the Knee Joint—Rapid Recovery

**Published:** 1872-09

**Authors:** Louis P. Griffin

**Affiliations:** 209½ Blue Island Avenue, Chicago


ARTICLE III.—*Lateral Dislocation of the Knee Joint—Rapid Recovery.* By LOUIS P. GRIFFIN, M.D., 209½ Blue Island Avenue, Chicago.

On August 10th, 1871, I was called in great haste to see Michael Foley, residing at 371 Catherine Street, reported to have been badly injured by a kick from a horse. Saw the patient a few moments after his receiving the injury.

Examination proved the case to be one of dislocation of the left knee joint laterally, uncomplicated with fracture or even the slightest abrasion of the skin. A faint redness of the skin, however, two inches below the outer side of the joint, was the only symptom of external injury. Chloroform was administered, and with the assistance of Drs. Hutchinson and Hamilton, by making contrary lateral pressure, at the same time with slight extension, the dislocation was readily reduced. The limb was then bandaged and a padded splint reaching from the hip to the external maleoli was applied to insure quiet. Cold water was then kept constantly applied to the joint by means of a small stream; no inflammatory symptoms made their appearance up to the 16th day, after which the water dressings were discontinued and passive motion of the joint commenced. August 27th, the patient had entirely recovered from the injury, and could use the limb without inconvenience. I am inclined to believe that the quick recovery was, in a great measure, due to the persistent use of the cold water dressings.

How many cases are there on record of lateral dislocation of the knee joint, where the patient recovered without leaving any traces of inconvenience in using the limb?

